# Adverse Drug Events by Sex After Adjusting for Baseline Rates of Drug Use

**DOI:** 10.1001/jamanetworkopen.2023.29074

**Published:** 2023-08-21

**Authors:** Tamara Rushovich, Annika Gompers, Jeffrey W. Lockhart, Ife Omidiran, Steven Worthington, Sarah S. Richardson, Katharine M. N. Lee

**Affiliations:** 1 Department of Social and Behavioral Sciences, Harvard T.H. Chan School of Public Health, Boston, Massachusetts; 2 Emory University Rollins School of Public Health, Atlanta, Georgia; 3 Department of Sociology, University of Chicago, Chicago, Illinois; 4 Harvard University, Cambridge, Massachusetts; 5 Institute for Quantitative Social Science, Harvard University, Cambridge, Massachusetts; 6 Harvard University, Cambridge, Massachusetts; 7 Tulane University, New Orleans, Louisiana

## Abstract

This cross-sectional study examines adverse drug events reported by sex in the US Food and Drug Administration (FDA) database after adjusting for drug use by males and females.

## Introduction

Although they have known limitations,^1^ spontaneous reporting pharmacovigilance databases like the US Food and Drug Administration (FDA) Adverse Event Reporting System (FAERS) and World Health Organization VigiBase are widely cited as evidence for claims that women experience adverse drug events (ADEs) at as high as twice the rate of men.^2,3^ Pharmacokinetics and pharmacodynamics are typically used to explain these sex differences^2^; however, many factors could influence the distribution of ADE reports by sex, including well-known disparities in the rates at which men and women use prescribed drugs.^4^ This study examined ADEs reported by sex in the FAERS database after adjusting for drug use by men and women. Information regarding how sex and gender are conceptualized in this study and its data sources appears in Supplement 1.

## Methods

This cross-sectional study used publicly available, deidentified data and was determined by the Harvard Institutional Review Board not to be research involving human participants. This study followed the Strengthening the Reporting of Observational Studies in Epidemiology (STROBE) reporting guideline.

**Figure 1.**
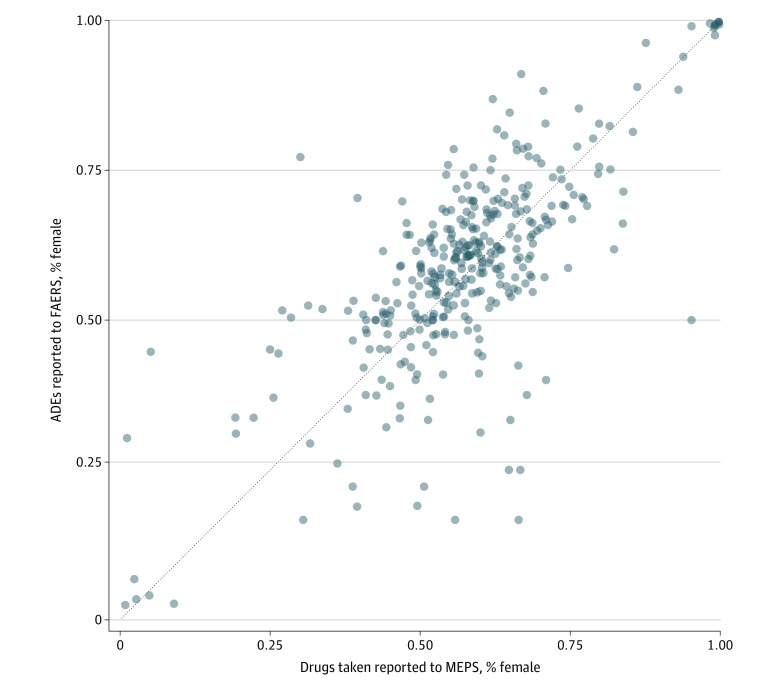
Correlation Between Sex Disparities in ADE Reports and Sex Disparities in Drug Use ADE reports by sex are highly correlated with sex disparities in rates of drug use (*r*, 0.74; 95% highest posterior density intervals, 0.70-0.78). Data are included for all 356 drugs that were in both data sets and where at least 50 people were observed. FAERS indicates US Food and Drug Administration Adverse Event Reporting System; MEPS, Medical Expenditure Panel Survey.

**Figure 2.**
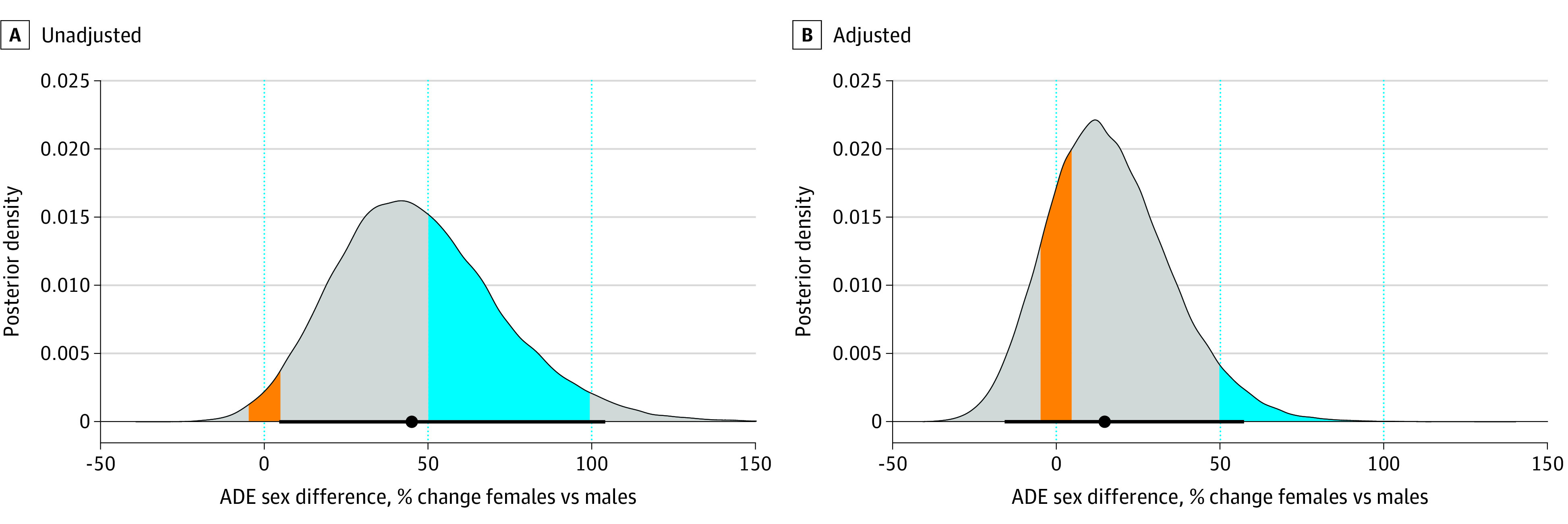
Posterior Distributions of Sex Differences in Reported Adverse Drug Events (ADEs) Before and After Adjustment for Drug Use The area under the curves that is shaded in orange represents the probability of a median sex disparity between −5% to 5%, while the area under the curves that is shaded in blue represents the probability of a median sex disparity between 50% to 100%. The black lines at the bottom of the curves represent 95% highest posterior density intervals, and the black points within those lines denote the medians of the posterior distributions. After adjustment for drug use (B), the probability of a median sex disparity between 50% to 100% reduces from 39.3% to 4.9%.

In this study, ADE data from FAERS were matched with drug use data from the Medical Expenditure Panel Survey (MEPS), a nationally representative survey of US health care use, for the years 2014 to 2019.^5^ Bayesian general linear models were used to estimate (1) the Pearson correlation coefficient (*r*) between the proportion of people using a drug who were females and proportion of ADE reports in FAERS for that drug filed by females (Figure 1) and (2) the association between the natural log of the number of ADE reports for a drug and the natural log of the number of people using that drug (eMethods in Supplement 1). Data management and analysis were conducted in Python software version 3.10.9 (Python Software Foundation) and R version 4.2.3 (R Project for Statistical Computing).

## Results

There were 19 940 532 ADEs reported in FAERs during the study, 12 622 357 (63.3%) of which were experienced by females. Reports in FAERS were highly correlated with between-sex variation in baseline rates of drug use (*r* = 0.74; 95% highest posterior density interval [HPDI], 0.70 to 0.78). In a model not adjusting for estimated drug use, the median number of reports of ADEs experienced by females was 45.1% (95% HPDI, 4.6% to 104.3%) higher than the number experienced by males. In a model adjusting for estimated drug use, the median number of reports of ADEs experienced by females reduced to 15.0% (95% HPDI, −15.8% to 57.6%) higher than that of males, a 3-fold decrease on average, and a sex difference of 0 became plausible (Figure 2).

With the unadjusted model, the probability of a median sex difference ranging from −5% to 5% (ie, close to 0) was 2.2%, whereas the probability of a sex difference in which the median number of ADEs is 50% to 100% higher for females compared with males was 39.3%. After adjusting for drug use, the probability of a sex difference close to 0 (ie, −5% to 5% higher median number of ADEs for females) was 17%, whereas a sex difference of 50% to 100% higher ADEs in females compared with males was reduced to 4.9% (Figure 2).

## Discussion

In this cross-sectional study, adjusting pharmacovigilance data from FAERS with nationally representative data on sex disparities in usage of drugs from MEPS greatly attenuated the apparent sex disparity in ADE reporting, reducing the gap in the median number of ADEs for women compared with men from 45.1% to 15.0%. The commonly cited claim that females experience 1.5 to 2 times the number of ADEs as males,^6^ was found to be highly unlikely, with a probability of less than 5% after accounting for drug usage. This study had limitations inherent to FAERS and MEPS data, including potential selection bias due to voluntary reporting, which prohibits the calculation of ADE incidence rates. Nevertheless, after accounting for underlying drug use, reported numbers of ADEs were similar between males and females when looking across drugs, suggesting that sex disparities in drug use may largely explain observed sex disparities in ADEs, bolstering evidence from a limited number of prior studies that have accounted for drug use in analyses of sex disparities in ADEs in pharmacovigilance data.^5^

## Notes

Supplement 1.
eMethods.

Supplement 2.
Data Sharing Statement

## References

[1] Center for Drug Evaluation and Research. Questions and answers on FDA’s Adverse Event Reporting System (FAERS). Food and Drug Administration. Published May 22, 2019. Accessed December 5, 2022. https://www.fda.gov/drugs/surveillance/questions-and-answers-fdas-adverse-event-reporting-system-faers

[2] Zucker I, Prendergast BJ. Sex differences in pharmacokinetics predict adverse drug reactions in women. Biol Sex Differ. 2020;11(1):32. doi:10.1186/s13293-020-00308-5 32503637 PMC7275616

[3] Tharpe N. Adverse drug reactions in women’s health care. J Midwifery Womens Health. 2011;56(3):205-213. doi:10.1111/j.1542-2011.2010.00050.x 21535369

[4] de Vries ST, Denig P, Ekhart C, . Sex differences in adverse drug reactions reported to the National Pharmacovigilance Centre in the Netherlands: an explorative observational study. Br J Clin Pharmacol. 2019;85(7):1507-1515. doi:10.1111/bcp.13923 30941789 PMC6595313

[5] Davis KE. Sample design of the 2020 Medical Expenditure Panel Survey Insurance Component. Agency for Health Care Research and Quality. 2021. Accessed July 13, 2023. https://www.meps.ahrq.gov/data_files/publications/mr34/mr34.pdf 38416859

[6] Martin RM, Biswas PN, Freemantle SN, Pearce GL, Mann RD. Age and sex distribution of suspected adverse drug reactions to newly marketed drugs in general practice in England: analysis of 48 cohort studies. Br J Clin Pharmacol. 1998;46(5):505-511. doi:10.1046/j.1365-2125.1998.00817.x 9833605 PMC1873702

