# Supplementary material for: Adverse Drug Events by Sex After Adjusting for Baseline Rates of Drug Use
Source: JAMA Netw Open. 2023 Aug 21;6(8):e2329074. doi: 10.1001/jamanetworkopen.2023.29074 (PMC10442708; doi:10.1001/jamanetworkopen.2023.29074)
Supplement: Supplement 1. — eMethods. [file jamanetwopen-e2329074-s001.pdf]

Supplemental Online Content

Rushovich T, Gompers A, Lockhart JW, et al. Adverse drug events by sex after adjusting for baseline rates of drug use. *JAMA Netw Open*. 2023;6(8):e2329074. doi:10.1001/jamanetworkopen.2023.29074

eMethods.

This supplemental material has been provided by the authors to give readers additional information about their work.

© 2023 Rushovich T et al. *JAMA Network Open.*

Detailed methods
Data sources

This analysis used publicly available, de-identified data and was determined not to be human subjects research (Harvard University IRB protocol number IRB22-0165).

Federal Adverse Event Reporting System (FAERS): Publicly available ADE reports from FAERS 2014 through 2019 were analyzed (Table 1). Each case reports a single primary suspect drug, which includes prescription and over-the-counter drugs, here referred to as “drug.” For this analysis, the standardized variable “product active ingredient” was used to match drugs. The analysis excluded cases where sex was listed as unknown (1,496, 0.018%), prefer not to say (158, 0.002%), intersex (109, 0.001%), or trans (73, 0.001%), as well as those with missing sex (809,859, 11.6%), resulting in 6,962,665 (3,770,878 female and 2,371,928 male) ADE reports. We did not exclude reports based on location (i.e. outside of the US) as it is not possible to know if the non-US reports are coming from non-US citizens or from US residents that were temporarily outside of the US. Additionally, FAERS is a US federal database and there are many non-US based adverse drug surveillance systems that a non-US citizen would probably be more likely to utilize than FAERS. This includes the European Medicines Agency’s EudraVigilance, The Canadian Network for Observational Drug Effect Studies (CNODES), the Asian Pharmacoepidemiology Network (AsPEN), the Australian Adverse Drug Reaction Reporting System, and the World Health Organization’s VigiBase.

Medical Expenditure Panel Survey (MEPS): MEPS is a nationally representative survey of healthcare utilization among US households, which includes questions about prescribed drugs obtained and used in the last year.[1,2] This includes over-the-counter drugs as well as those available only by prescription. These data were weighted by education, census region,

Metropolitan Statistical Area status, race/ethnicity, sex, and age to produce nationally representative estimates of prescribed drug use in the US population. To align with the data available in FAERS, MEPS data from 2014 - 2019 were pooled using the {survey} package in R[3] and the survey weights provided by MEPS.[4]

The term "sex" here refers to groups of individuals classified as men/males and women/females in FAERS and MEPS, though social, gendered factors may drive differences between groups rather than biological sex alone. The term "gender/sex" indicates variation between and among men/males and women/females when the contributing role of gender- and sex-related factors is unknown or may not be easily disentangled.[5]

Data Analysis

In the FAERS analysis, only drugs with at least 50 ADE reports were used. For analyses using MEPS, only drugs with at least 50 survey responses were used. In analyses that use both FAERS and MEPS, only the years and drugs present in both data sets were used. Number of reports was used as the unit of analysis across data sets (rather than number of drugs or number of ADEs, as a report from one person may include multiple drugs and/or ADEs). FAERS data were stratified by drug, preventing very common or very rare drugs from disproportionately influencing results.

For all analyses, we employed a Bayesian approach to statistical inference for two reasons:

1. Applied researchers often find Bayesian quantities of interest easier to interpret than their frequentist counterparts.[6] In fact, this is so widespread that researchers sometimes interpret frequentist confidence intervals and p-values incorrectly as if they were Bayesian credible intervals and posterior probabilities.[7] For example, a 95% confidence interval is typically misinterpreted as meaning there is a 95% probability that a given

© 2023 Rushovich T et al. *JAMA Network Open.*

population parameter resides between the upper and lower limit, when in fact only a Bayesian credible interval allows this interpretation.[8]

2. We wish to make probabilistic statements about the location of population parameter values. For example, it has been stated in the literature that ADEs are 1.5 to 2 times more likely in females compared with males. Using Bayesian inference, we can calculate the probability that this hypothesis is true, given our model and data.[7] In contrast, frequentist inference does not allow for probabilistic statements about parameters, because they are assumed to be fixed while data are random.

The general linear model specifications used in our analyses were as follows:

1. Correlation model: $std(FAERS\ prop) = 0 + \beta_1 \times std(MEPS\ prop) + \varepsilon$
2. ADE unadjusted model: $log\ (n\ FAERS) = \beta_0 + \beta_1 \times\ sex + \varepsilon$
3. ADE adjusted model: $log\ (n\ FAERS)\ = \beta_0 + \beta_1 \times\ sex + \beta_2 \times log\ (n\ MEPS)\ + \varepsilon$

For the correlation model, “FAERS prop” and “MEPS prop” are the proportion of ADE reports in FAERS for a given drug filed by women and the proportion of people using that drug who were women, respectively (Figure 1). Both variables were standardized by mean-centering and dividing by their sample standard deviations prior to inclusion in the model. This correlation shows the extent to which variation in drug use by sex correlated with variation in ADEs by sex reported to FAERS. For the ADE models, “n FAERS” and “n MEPS” are the number of ADE reports for a given drug and the number of people using that drug, respectively. Both variables were natural log transformed since they exhibited severe right skew (some drugs were much more common than others). The “sex” term is a binary indicator variable coded as male=0 and female=1. In each model, the parameter of interest is β_1, which in the correlation model is the Pearson correlation coefficient and in the two ADE models is the percentage median change in

© 2023 Rushovich T et al. *JAMA Network Open.*

ADE reports for women compared to men, with and without adjusting for the number of people taking each drug.

We report effect size point estimates as medians of the posterior distribution and effect size uncertainty as 95% highest posterior density intervals (i.e., the interval within which an unobserved parameter value falls with 95% probability) as well as posterior probabilities of directional hypotheses (i.e., the probability that a hypothesis is supported, given the model and data). All effect size estimates are based on 100,000 post-warmup posterior iterations after 10,000 warmup iterations. For each model, we used a Gaussian likelihood with identity link function and weakly informative priors for all location effects parameters (Student's t distribution with 5 degrees of freedom, a mean of 0, and standard deviation of 2.5) and correlation parameters (Lewandowski-Kurowicka-Joe distribution with η equal to 1) and confirmed that convergence to a stable posterior distribution had been achieved using four independent Markov chains with a Gelman-Rubin R-hat value criterion of 1.0.[9] The use of weakly informative priors reflected our diffuse a priori understanding of effect locations and our desire to let the data dictate the posterior estimates of these effects. Model goodness of fit was determined using posterior predictive checks with 1000 simulations from the model. All models were fitted with the R package {brms}.[10]

Limitations

This study has several limitations. The FAERS database has a high level of missingness for key data including weight and age, preventing those variables from being included in this analysis. The FAERS dataset has a moderate amount of missingness for the sex variable. FAERS data also likely has a high degree of selection bias due to the nature of voluntary reporting. Despite this, pharmacovigilance databases such as FAERS are widely referenced as the basis of estimates of sex disparities in ADEs. Because there is no comprehensive national database or national health record system of drug usage in the U.S., drug use was estimated

© 2023 Rushovich T et al. *JAMA Network Open.*

using MEPS, which is subject to known limitations, including the potential for recall bias.[1] Replicating the analysis in vaccine passive surveillance systems (e.g. VAERS) and using multiple alternative sources of data on drug use by gender/sex, such as state-level controlled substance reporting, will make possible more robust inferences about the relationship between rates of drug use and rates of ADE reports.

References

1. Agency for Healthcare Research and Quality (AHRQ). Data Overview. Medical Expenditure Panel Survey. Published April 22, 2019. Accessed January 10, 2023. https://www.meps.ahrq.gov/mepsweb/data_stats/data_overview.jsp
2. Davis KE. Sample design of the 2020 Medical Expenditure Panel Survey Insurance Component. *Agency for Health Care Research and Quality*. 2021;Methodology Report #34. https://www.meps.ahrq.gov/data_files/publications/mr34/mr34.pdf
3. Lumley T. Survey: Analysis of Complex Survey Samples. Published online July 19, 2021. Accessed January 19, 2023. https://CRAN.R-project.org/package=survey
4. Mitchell E. Analyzing MEPS data using R. GitHub. Published November 18, 2022. Accessed January 10, 2023. https://github.com/HHS-AHRQ/MEPS
5. van Anders SM. Beyond sexual orientation: Integrating gender/sex and diverse sexualities via Sexual Configurations Theory. *Arch Sex Behav*. 2015;44(5):1177-1213. doi:10.1007/s10508-015-0490-8
6. Kruschke JK, Liddell TM. Bayesian data analysis for newcomers. *Psychon Bull Rev*. 2018;25(1):155-177. doi:10.3758/s13423-017-1272-1
7. Kruschke JK, Liddell TM. The Bayesian New Statistics: Hypothesis testing, estimation, meta-analysis, and power analysis from a Bayesian perspective. *Psychon Bull Rev*. 2018;25(1):178-206. doi:10.3758/s13423-016-1221-4
8. Morey RD, Hoekstra R, Rouder JN, Lee MD, Wagenmakers EJ. The fallacy of placing confidence in confidence intervals. *Psychon Bull Rev*. 2016;23(1):103-123. doi:10.3758/s13423-015-0947-8
9. Gelman A, Rubin DB. Inference from Iterative Simulation Using Multiple Sequences. *Statist Sci*. 1992;7(4). doi:10.1214/ss/1177011136
10. Bürkner PC, Gabry J, Weber S, et al. brms: Bayesian regression models using “Stan.” Published online September 19, 2022. Accessed January 10, 2023. https://CRAN.R-project.org/package=brms

© 2023 Rushovich T et al. *JAMA Network Open.*
