# Supplementary material for: Adverse Drug Events by Sex After Adjusting for Baseline Rates of Drug Use
Source: JAMA Netw Open. 2023 Aug 21;6(8):e2329074. doi: 10.1001/jamanetworkopen.2023.29074 (PMC10442708; doi:10.1001/jamanetworkopen.2023.29074)
Supplement: Supplement 2. — Data Sharing Statement [file jamanetwopen-e2329074-s002.pdf]

Data Sharing Statement

Rushovich. Adverse Drug Events by Sex After Adjusting for Baseline Rates of Drug Use. *JAMA Netw Open*. Published August 21, 2023. doi:10.1001/jamanetworkopen.2023.29074

Data
Data available: Yes
Data types: Deidentified participant data
How to access data: All the data for this study are publicly available on the FAERS and MEPS websites.
When available: With publication

Supporting Documents
Document types: None

Additional Information
Who can access the data: Data are publicly available
Types of analyses: Data are publicly available
Mechanisms of data availability: Data are publicly available
Any additional restrictions: None
